# A Randomized Clinical Trial Comparing the Efficacy of Melatonin and Amitriptyline in Migraine Prophylaxis of Children

**Published:** 2018

**Authors:** Razieh FALLAH, Fatemeh FAZELISHOROKI, Leila SEKHAVAT

**Affiliations:** 1 Department of Pediatrics, Growth Disorders of Children Research Center, Shahid Sadoughi University of Medical Sciences, Yazd, Iran.; 2 Department of Obstetrics and Gynecology, Shahid Sadoughi University of Medical Sciences, Yazd, Iran.

**Keywords:** Migraine, Child, Prevention, Amitriptyline, Melatonin

## Abstract

**Objective:**

The aim of the present research was to compare the effectiveness and tolerability of melatonin and amitriptyline in pediatric migraine prevention.

**Materials & Methods:**

In a parallel single-blinded randomized clinical trial, 5-15 yr old children with diagnosis of migraine that preventive therapy was indicated in whom and were referred to Pediatric Neurology Clinic of Shahid Sadoughi Medical Sciences University, Yazd-Iran from 2013-2014, were randomly allocated to receive 1 mg/kg amitriptyline or 0.3 mg/kg melatonin for three consecutive months.

**Results:**

Forty one girls (51.3%) and 39 boys (48.7%) with mean age of 10.44±2.26 yr were evaluated. Good response was seen in 82.5% of amitriptyline and 62%.5 of melatonin groups and amitriptyline was statistically significant more effective (*P*=0.04). Severity, duration and Pediatric Migraine Disability Assessment score (PedMIDAS) of headache reduced with melatonin from 6.05±1.63 to 4.03±1.54 scores, from 2.06±1.18 to 1.41± 0.41 hours, and from 33.13±9.17 to 23.38±9.51 scores, respectively. Severity, duration and PedMIDAS of headache decreased with amitriptyline from 6.41±1.67to 2.25±1.21, from 2.55 ±1.85to 0.56±0.51h, and from 31.4±9.33 to 8.28 ± 3.75, respectively (All *P* < 0.05). Both drugs were effective in reduction of monthly frequency, severity, duration and disability of headache. Daily sleepiness was seen in 7.5% of melatonin group as a side effect and adverse events were seen in 22.5% of amitriptyline group including daily sleepiness in four, constipation in three and fatigue in two children and melatonin was safer than amitriptyline (value = 0.04).

**Conclusion:**

Amitriptyline and melatonin are effective and safe in pediatric migraine prophylaxis but amitriptyline can be considered as a more effective drug.

## Introduction

Migraine is one of the most common health problems of children and up to 28% of older teenagers have migraine headache (1). Up to one-third of migraineurs children have criteria for drugs prophylaxis. Migraine prevention therapy should be prescribed if headaches occur more than 3-4 times per month to reduce its frequency to 1-2 attacks or less per month or if headache disrupts daily kid function to decrease headaches disability to achieve Pediatric Migraine Disability Assessment score (pedMIDAS) less than 10 (2).

Antihistamines, antihypertensive, antidepressants and antiepileptic drugs have been consumed in pediatric migraine prophylaxis. However, a standardized and unique guideline for migraine prevention in children does not exist and flunarizine and recently topiramate have been approved by Food and Drug Administration in pediatric population and other randomized clinical trials for the use of preventive drugs in migraine of children are fiercely needed (2, 3). 

Amitriptyline as tricyclic antidepressants with minimal side effects is the most common migraine preventive drug in children, but amitriptyline should not be used in children with rapid or irregular heartbeat and prolonged QT syndrome and orderly electrocardiograms monitoring might be required (4). 

"Melatonin (N-acetyl-5-methoxytryptamine) is an indole compound which is mainly produced in the pineal gland from tryptophan amino acid and secreted into the blood and cerebrospinal fluid and its main role is modulation of the circadian rhythm of sleep .The level of melatonin might be decreased in migraine headaches and it may play an important role in migraine pathogenesis. This chronobiotic agent has been used as an effective drug in treatment of migraine headaches. Possible mechanisms of melatonin in treatment of headache might be related to its anti-inflammatory, hypnotic, analgesic, antioxidative and toxic free radical scavenging effects, decrease of pro-inflammatory cytokine upregulation, nitric oxide synthase activity and inhibition of dopamine release, stabilizing of membrane, glutamate neurotoxicity protection and neurovascular regulation" (5).

Effectiveness of three-milligram oral melatonin at bedtime in 14 children with recurrent migraine headache, aged 6-16 yr, is reported (6).

We aimed to test the hypothesis that whether or not melatonin was superior to amitriptyline for migraine prophylaxis in 5-15 yr-old children suffer frequent or disabling headaches referred to Pediatric Neurology Clinic. We wanted to compare the effectiveness and tolerability of amitriptyline and melatonin for pediatric migraine prevention. 

## Materials and Methods

In a randomized single-blind clinical, open-label, parallel group study, effectiveness and tolerability of amitriptyline and melatonin for migraine prophylaxis in 5-15 yr old children who had migraines with and without aura, in whom preventive therapy was indicated and were referred to Pediatric Neurology Clinic of Shahid Sadoughi Medical Sciences University, Yazd, Iran from June 2013 to January 2014, were evaluated.

Sample size based on Z formula and confidence interval of 95% with 80% power, type one error of 5%, good response (more than 50% decrease in monthly headache frequency during the follow-up period) of 70% for melatonin in pilot study (5) and an effect size (difference in frequency of good response between the two groups) of 30% for the primary endpoint, was assessed in 40 children per group.

Informed consent was taken from patients’^,^ parents before administration of the drugs. The study was approved by the Ethics Committee of Shahid Sadoughi University of Medical Sciences, Yazd, Iran. The researchers were not funded by the drugs companies. This study is registered in Iranian clinical trials with registration number: IRCT201305292639N12. 

Inclusion criteria included children aged 5-15 yr, having migraine headache (with or without aura) based on second edition of the International Classification of Headache Disorders criteria (7) in clinical evaluation by a pediatric neurologist, having one or more headache attack per week, having moderate or severe headache disability PedMIDAS more than 20, and not used any migraine preventive therapy. 

The children with secondary headaches or systemic illness (renal or hepatic insufficiency, cardiac, hematologic or endocrine diseases) based on physical exam and paraclinical evaluation (laboratory assessment or neuroimaging in suspected to while increased intracranial pressure) and children who did not full the three months treatment period were excluded.

The trial used computer generated equal simple randomization by random numbers and allocation ratio was 1:1 for the two groups. Randomization and blinding was done by an investigator with no clinical involvement in the trial. Participants, data gatherers, outcome valuators, data analysts and the nurse who handed over the intervention, were all kept blinded to the allocation. The medications were handed over by the clinic nurse and each was packaged and labeled according to the drug code schedule that was created before the study. The nurse opened the package that included the amount of the drug in one tablet and its^, ^dosage and then was rendered to the research pediatric neurologist who had calculated the drug dosage based on the weight of the child. Primary and secondary endpoints were assessed by the resident of research who was not informed of the group allocation (8).

The children were randomly allocated to two groups to receive 0.3 mg/kg melatonin (maximum 6 mg) or 1mg/kg/day amitriptyline (maximum 50 mg) for 90 consecutive days. In both groups, the drugs were administered orally in single dose at bedtime. Participants of the study were seen by the pediatric resident of research in Pediatric Neurology Clinic of Shahid Sadoughi Hospital every two weeks for three consecutive months. Their parents were asked about frequency, severity, duration and disability score of headaches (9), frequency and severity clinical adverse effects of the drugs, number of analgesics and repots of their parents in diaries were reviewed. The researchers had no information about life style modification or herbal and traditional medicine during the follow up period. Measurement equipment of amitriptyline and melatonin blood level were not accessible in our center.

Safety assessment contained immediate reports of adverse events, physical examination and evaluation of vital signs. Children parents were asked to call the researcher immediately if severe and critical side effects such as faint and cardiac arrhythmia, seizure, severe daytime sleepiness, agitation and excitability, mood and behavioral changes, severe skin eruptions, severe constipation and hypothermia would happen.

Laboratory assessment (hepatic, renal, hematologic and coagulation function) was done for all participants at screening visits and in the children who suffered adverse events during the treatment period.

 The participants were allowed to use nonsteroidal anti-inflammatory analgesics (acetaminophen or ibuprofen) for alleviation of moderate to severe headache attacks during the research period.

"Severity of headache was assessed by asking each child to grade majority of headache pain on visual analogue scale or VAS (10) on 10-point scale as no pain = scale of 0 and the most severe pain = 10. AVAS is a horizontal or vertical 10 cm long line, marked at the extremes with “no pain and worst pain imaginable”. The children were asked to place a mark on the line that represented their pain level. The drugs were continued for 90 consecutive days and then, monthly frequency, severity and duration of headache and pedMIDAS of before and after three months of drugs usage, were compared. More than 50% of reduction in monthly headache frequency was considered as good response "(5).

Primary endpoints were frequency of good response (more than 50% of reduction in monthly headache frequency) and efficacy in reduction of severity, duration and disability of headache. Secondary outcome was drugs clinical side effects.

The data were analyzed using SPSS: 15 statistical software (Chicago, IL, USA). Chi-square test or Fisher exact test was used for data analysis of qualitative variables and mean values were compared by *t*-test. Differences were considered significant at *P* values of less than 0.05.

## Results

Three children in melatonin group and four patients in amitriptyline group loosed to follows: up and two patients in melatonin group and two in amitriptyline discontinued drug usage during follow up period. Finally, 41girls (51.3%) and 39 boys (48.7%) with mean age of 10.44 ± 2.26 yr were evaluated.

Comparison of some characteristics of the children in both groups is shown in Table 1 which indicates that age, onset age of migraine, monthly frequency, severity, duration and disability of headache, number of analgesics usage, sex distribution, type of migraine and positive family history of migraine were not statistically significant different in both groups.

After three months of treatment, good response (more than 50% reduction in monthly headache frequency) was seen in 25 patients (62.5%) of melatonin (95% confidence interval: 0.45–0.71) and in 33 (82.5%) of amitriptyline group (95% confidence interval: 0.75 –0.93) and amitriptyline was statistically significantly more effective (*P*=0.04).

Comparison of headache characteristics before and after treatment in melatonin and amitriptyline groups is presented in Table 2 and Table 3 that shows both drugs were effective in reduction of monthly frequency, severity, duration and disability of headache.

Table 4 shows comparison of headache characteristics after treatment, which indicates that amitriptyline, was more effective than melatonin in reduction of monthly frequency, severity, duration and disability of headache. However, number of analgesic usage was not statistically significant different in melatonin and amitriptyline groups.

Daily sleepiness, as a side effect, was seen in 7.5% (N=3) of melatonin group. In 22.5% (N=9) of amitriptyline group, adverse events were seen including daily sleepiness in four, constipation in three and fatigue and malaise in two children. The adverse events were more frequent in amitriptyline group (value = 0.04).

Excessive daytime sleepiness as a serious side effect, which caused to stop the drug use, was seen in two children in each group.

## Discussion

Varieties of medications have been used for treatment and prevention of migraine in pediatric population. In the present research, effectiveness and tolerability of amitriptyline and melatonin for pediatric migraine prophylaxis in 5-15 yr old children were compared. The results showed that both drugs were effective, but amitriptyline was more effective and melatonin was safer than amitriptyline.

**Table 1:** Comparison of some characteristics of children in both groups

Group		Melatonin	Amitriptyline	*P*. value	Statistical test that was used
Data					
Age in years (mean ± SD)		10.57 ± 2.44	10.11 ± 2.13	0.4	Independent *t*-test
Onset age of migraine (mean ± SD)		8.59 ± 1.56	8.34 ± 2.45	0.6	Independent *t*-test
Monthly headache frequency (mean ±SD)		16.7 ± 6.68	15.8 ± 8.49	0.09	Independent *t*-test
Severity of headache (mean ±SD)		6.05 ± 1.63	6.41 ± 1.67	0.4	Independent *t*-test
Headache duration in hours (mean ±SD)		2.06 ± 1.18	2.55 ± 1.85	0.2	Independent *t*-test
Headache disability: pedMIDAS (mean ±SD)		33.13 ± 9.17	31.4 ± 9.33	0.4	Independent *t*-test
Number of analgesic(Acetaminophen or Ibuprofen ) usage during follow up period (mean ± SD)		12.32±3.9	13.24±2.6	0.8	Independent *t*-test
Sex	Girl	22	19	0.5	Chi-square test
	Boy	18	21		
Type of migraine	Without aura	23	29	0.2	Chi-square test
	With aura	17	11		
Positive family history of migraine	Yes	35	33	0.9	Chi-square test
	No	5	7		

**Table 2:** Comparison of headache characteristics before and after treatment in melatonin group

Group	Before treatment (mean ±SD)	After treatment (mean ±SD)	*P*. value	Statistical test that was used
Data				
Monthly headache frequency	16.7 ± 6.68	9.03 ± 4.47	0.001	Independent *t*-test
Severity of headache	6.05 ± 1.63	4.03 ± 1.54	0.01	Independent *t*-test
Headache duration in hours	2.06 ± 1.18	1.41 ± 0.41	0.001	Independent *t*-test
Headache disability: pedMIDAS	33.13 ± 9.17	23.38 ± 9.51	0.001	Independent *t*-test
Number of analgesic usage during follow up period	12.32±3.9	7.22±2.81	0.01	Independent *t*-test

**Table 3:** Comparison of headache characteristics before and after treatment in amitriptyline group

Group	Before treatment (mean ±SD)	After treatment (mean ±SD)	*P*. value	Statistical test that was used
Data				
Monthly headache frequency	15.8 ± 8.49	4.28 ± 2.68	0.001	Independent *t*-test
Severity of headache	6.41 ± 1.67	2.25 ± 1.21	0.0001	Independent *t*-test
Headache duration in hours	2.55 ± 1.85	0.56 ± 0.51	0.001	Independent *t*-test
Headache disability: pedMIDAS	31.4 ± 9.33	8.28 ± 3.75	0.0001	Independent *t*-test
Number of analgesic usage during follow up period	13.24±2.6	6.11±2.7	0.001	Independent *t*-test

**Table 4:** Comparison of headache characteristics after treatment in both groups

Group	Melatonin (mean ±SD)	Amitriptyline (mean ±SD)	*P*. value	Statistical test that was used
Data				
Monthly headache frequency	9.03 ± 4.47	4.28 ± 2.68	0.001	Independent *t*-test
Severity of headache	4.03 ± 1.54	2.25 ± 1.21	0.0001	Independent *t*-test
Headache duration in hours	1.41 ± 0.41	0.56 ± 0.51	0.0001	Independent *t*-test
Headache disability: pedMIDAS	23.38 ± 9.51	8.28 ± 3.75	0.0001	Independent *t*-test
Number of analgesic usage during follow up period	7.22±2.81	6.11±2.7	0.5	Independent *t*-test

In 18-65 yr old migraineurs in Brazil, melatonin and amitriptyline in comparison with placebo-decreased frequency of migraine headache and tolerability of melatonin was more than amitriptyline (11). Effectiveness of melatonin in reduction of monthly frequency, severity, duration and disability of migraine headache of children in present research was in compliance to another study (6). 

In this study, more than 50% reduction in monthly headache frequency was seen in 62.5% of children in melatonin group. However, the rate of good response had been 58% in Italian study (6) and 75% in Brazilian study (11). A possible explanation for this discrepancy is differences in age, drug dosage, race, sample size and design of the study.

Headache and sleep are related interdependently, lack of sleep and excessive sleep can both cause migraine (12) and melatonin irregular production has some links to sleep disorders, linked to headache. Interruption of night sleep due to headache might cause sleeplessness and daytime drowsiness and effectiveness of melatonin in control of migraine headache might be related to normalization and adjustment of sleep circadian rhythm (13). 

In the present study, melatonin was tolerated well and no life-threatening clinical side effects were seen in kids and safety of melatonin similar to other researches (6, 14, 15). The main side effect was daytime sleepiness as in Sao Paulo, Brazil (11).

Side effects including worsening sleep pattern, agitation, behavioral change, hyperactivity, seizure, nightmares, constipation, nightmares, hypotension and sleep disorders were reported (5, 11, 16, 17).

In the present research, 82.5% of patients in amitriptyline group showed good response. Positive response rate for amitriptyline was also 89% in Lewis et al. study (18), 84.2% in Hershey et al. study (19) and 62% in the study of Kalita et al study in adults (20). In Dhaka, Bangladesh, amitriptyline was effective and with minimal side effects in prevention of migraine (21). However, in the USA, amitriptyline was more effective than placebo in prevention of migraine in 8 weeks but not in 12, 16, or 20 weeks (22).

In this study, 22.5% of children in amitriptyline group showed side effects such as daily sleepiness, constipation, malaise, and majority of them tolerated amitriptyline well and without life- threatening side effects. However, other adverse events such as dry mouth, dry eyes, lightheadedness and cardiac arrhythmia have been reported (23).

Prophylactic migraine therapy should be considered in children with frequent and functional disabling headaches. Parents and caregivers must learn about different options of migraine prophylaxis including pharmacologic and non-pharmacological behavioral techniques. In infants and younger kids who cannot swallow tablets, cyproheptadine might be superior. Amitriptyline as one of tricyclic antidepressants has been used as an effective drug for the prevention of migraine headache in children since 1970 and is preferable because of its once daily dosing and minimal side effects. In comorbid situations such as migraine and depression, amitriptyline might be more beneficial and abundance of amitriptyline adverse events such as sleepiness can be decreased by low dosage prescription (23- 25).

The limitations of the present study were lack of placebo, short duration of treatment and not following up the patients after discontinuation of the treatment. 

**In conclusion, **results of this research can be viewed as promising for it showed that amitriptyline and melatonin are effective and without life-threatening adverse events in pediatric migraine prophylaxis but amitriptyline was superior to melatonin and melatonin was tolerated better.
